# Proteolytic lactic acid bacteria for food safety and One Health: Degradation of cow’s milk allergens and prospects for hypoallergenic dairy development

**DOI:** 10.14202/vetworld.2025.2918-2933

**Published:** 2025-09-30

**Authors:** Chong Wang, Linglin Fu, Joo Shun Tan, L. Ravithej Singh, Julalak Chuprom

**Affiliations:** 1 Food Safety Key Laboratory of Zhejiang Province, School of Food Science and Biotechnology, Zhejiang Gongshang University, Hangzhou 310018, China; 2 Division of Bioprocess Technology, School of Industrial Technology, Universiti Sains Malaysia, Gelugor, 11800, Penang, Malaysia; 3 Division of Fluoro-Agrochemicals, CSIR - Indian Institute of Chemical Technology, Tarnaka, Hyderabad, India; 4 Division of Chemical Sciences, Academy of Scientific and Innovative Research, Ghaziabad, India; 5 Department of General Education, School of Languages and General Education, Walailak University, 222, Thaiburi, Thasala, Nakhon Si Thammarat, 80160, Thailand

**Keywords:** cow’s milk allergy, food safety, lactic acid bacteria, One Health, probiotics, proteolytic activity

## Abstract

**Background and Aim:**

Cow’s milk allergy (CMA) is one of the most common food allergies, particularly in infants and young children, caused mainly by β-lactoglobulin (β-LG) and caseins. Conventional methods to reduce milk allergenicity, including heat and pressure treatments, often compromise nutritional quality or lack industrial feasibility. Safe, natural approaches to allergen reduction are essential for both food safety and One Health perspectives, as CMA contributes to nutritional deficiencies and impacts global health. This study aimed to isolate and characterize proteolytic lactic acid bacteria (LAB) from Chinese dairy products and fermented foods, evaluate their ability to degrade major cow’s milk allergens, and assess their probiotic and safety profiles for application in hypoallergenic dairy products.

**Materials and Methods:**

Seventy-six LAB isolates were obtained from dairy and fermented foods and screened for proteolytic activity using skim milk agar and sodium dodecyl sulfate-polyacrylamide gel electrophoresis. The most active isolates were identified by phenotypic characterization and 16S ribosomal RNA sequencing. Probiotic potential was evaluated through in vitro gastrointestinal tolerance, bile salt hydrolase (BSH) activity, antimicrobial activity, and antibiotic susceptibility. Safety was assessed through hemolytic activity and screening for virulence-associated genes.

**Results:**

Seventy isolates exhibited proteolytic activity, of which 7 (S30, S44, S46, S52, S63, S67, and S76) showed strong hydrolysis of β-LG and β-casein. These were identified as *Streptococcus thermophilus*, *Lactobacillus fermentum*, *Lactobacillus plantarum*, *Lactobacillus casei*, *Lactobacillus rhamnosus*, and *Lactobacillus paracasei*. Notably, *L. rhamnosus* S46 achieved complete degradation of β-LG while maintaining high survival (>83%) under simulated gastrointestinal conditions, with BSH activity and broad antimicrobial effects. Most isolates lacked virulence genes and hemolytic activity, except *L. paracasei* S67.

**Conclusion:**

Proteolytic LAB strains, particularly *L. rhamnosus* S46 and *L. plantarum* S52, exhibited strong allergen-degrading activity, probiotic potential, and safety profiles, supporting their application in hypoallergenic dairy production. From a food safety and One Health perspective, these strains represent natural, functional alternatives for reducing milk allergenicity, improving consumer health, and supporting sustainable dairy innovation. However, *in vivo* validation and pilot-scale trials in real dairy systems are necessary to confirm industrial feasibility and consumer acceptance.

## INTRODUCTION

Food allergy represents a major public health challenge, affecting approximately 5%–8% of children and 1%–2% of adults worldwide. According to the World Allergy Organization, an estimated 220–250 million people suffer from food allergies globally [1]. Among various allergenic foods, eight, collectively termed the “Big 8,” account for over 90% of cases. These include cow’s milk, eggs, peanuts, tree nuts, soybeans, fish, and shellfish [2], with cow’s milk being the most prevalent allergen [3].

Cow’s milk allergy (CMA) is particularly common in early childhood, with a reported prevalence of 0.5%–3% in 1-year-old children in developed countries [4]. The principal allergenic proteins in milk are caseins (αS1-casein, αS2-casein, and β-casein [β-CN]) and whey proteins (α-lactalbumin [α-LA] and β-lactoglobulin [β-LG]), both of which are recognized as major allergens [5]. Given the widespread nutritional and industrial use of cow’s milk, complete avoidance is difficult and may lead to growth impairment and nutrient deficiencies in children. Thus, strategies to reduce or modify the allergenic potential of milk proteins are urgently needed.

Several technological interventions have been investigated. Heat treatment is widely used but often diminishes the nutritional quality of proteins while only partially reducing allergenicity [6]. High-pressure processing alters protein structures, but it faces limitations in terms of cost and commercial scalability [7]. In contrast, lactic acid fermentation, one of the oldest bioprocessing methods, offers a promising natural approach. Lactic acid bacteria (LAB) utilize proteolytic systems to hydrolyze milk proteins during fermentation, thereby enhancing digestibility, sensory quality, and nutritional value, while generating bioactive peptides that are beneficial to human health [8].

The LAB proteolytic system consists of (i) cell wall proteinases that cleave caseins into oligopeptides, (ii) transport systems that internalize peptides, and (iii) intracellular peptidases that degrade them into amino acids [9]. This enzymatic breakdown can disrupt antigenic epitopes, reducing allergenicity. Furthermore, consumer demand for functional foods has accelerated the incorporation of probiotics, defined as “living microorganisms that confer health benefits when consumed in adequate amounts” [10], into dairy products. A previous study by Ye *et al*. [11] confirms that certain LAB strains can lower milk allergenicity, although the extent of reduction is strain-dependent and influenced by fermentation conditions [12].

Despite progress in allergen reduction techniques, current approaches such as heat treatment and high-pressure processing remain limited by their negative impact on nutritional quality or lack of commercial feasibility [6, 7]. Lactic acid fermentation has emerged as a promising natural method, yet the effectiveness of allergen degradation is highly strain-dependent and influenced by fermentation conditions [11, 12]. Critically, few studies have systematically investigated LAB strains that combine strong proteolytic capacity against major allergens (particularly #x03B2;-LG) with probiotic resilience (gastrointestinal survival, bile salt hydrolase [BSH] activity, and antimicrobial effects) and comprehensive safety profiles (absence of virulence genes and hemolytic activity). The lack of such integrative evaluations limits the identification of robust LAB candidates for safe, hypoallergenic dairy product development within a food safety and One Health context.

This study aimed to isolate and characterize proteolytic LAB strains from traditional Chinese dairy products and fermented foods. The specific objectives were to:

1. Screen isolates for proteolytic activity and their ability to degrade major milk allergens, including β-LG and β-CN.
2. Evaluate probiotic traits, including gastrointestinal tolerance, BSH activity, and antimicrobial effect.
3. Assess safety aspects through detection of virulence-associated genes, hemolytic activity, and antibiotic susceptibility.

By integrating these assessments, the study sought to identify safe, functional LAB strains capable of reducing cow’s milk allergenicity, thereby supporting the development of hypoallergenic, probiotic-enriched dairy products aligned with food safety and One Health principles.

## MATERIALS AND METHODS

### Ethical approval

This study did not involve human participants, human samples, or animal experiments; therefore, ethical approval was not required. The probiotic strains used were isolated from dairy products and fermented foods, and all laboratory work was conducted in compliance with the biosafety regulations of the Food Safety Key Laboratory of Zhejiang Province, School of Food Science and Biotechnology, Zhejiang Gongshang University.

### Study period and location

This study was conducted from March 2022 to February 2023 at the Food Safety Key Laboratory of Zhejiang Province, School of Food Science and Biotechnology, Zhejiang Gongshang University.

### Chemicals and media

Allergen proteins from bovine milk, including β-LG, α-LA, β-CN, and α-casein, were purchased from Sigma Chemical Co. (St. Louis, MO, USA). M17 and De Man, Rogosa and Sharp (MRS) media were obtained from Qingdao Hope Bio-Technology Co., Ltd. (Qingdao, China). Non-fat powdered milk was obtained from BBI Life Sciences Corporation (Shanghai, China). All the chemicals used in the experiments were of analytical grade.

### Isolation and preliminary LAB identification

LAB strains were isolated from various dairy products. Samples were collected from various markets across China. Each 10 mL or 10 g sample was aseptically homogenized with 90 mL of phosphate-buffered saline (PBS) (PBS; 50 mM, pH 7.0) for 2 min using a stomacher (EASY MIX, AES Chemunex, Rennes, France). Ten-fold serial dilutions were prepared in PBS solution (50 mM, pH 7.0), and 0.1 mL aliquots were spread onto MRS and M17 agar plates supplemented with 0.004% (w/v) bromocresol purple (Sigma). All plates were incubated at 37°C for 24–72 h under aerobic and anaerobic conditions. To create anaerobic conditions, anaerobic jars (GasPak System-Oxoid, Basingstoke, Hampshire, England) were used in combination with AnaeroGen 2.5 L sachets and indicator strips (Oxoid Thermo Fisher Scientific, Waltham, USA). Yellow colonies with distinct morphologies (shape, size, and surface texture) were selected and further purified using another agar plate containing the same culture medium and conditions [13]. To confirm their identity, all LAB isolates were subjected to Gram staining and catalase testing. Catalase activity was detected by placing drops of 3% (v/v) hydrogen peroxide (H_2_O_2_) solution on cultures, and the immediate formation of bubbles indicated the presence of catalase in the cells. Only Gram-positive and catalase-negative strains were selected and subcultured in MRS or M17 broth containing 0.05% (w/v) L-cysteine (Sigma). The selected strains were stored in 20% (v/v) glycerol at −20°C.

### Confirmation of proteolytic activity

LAB strains were initially screened for proteolytic enzyme production using a qualitative spotting method. Selected strains were inoculated onto skim milk agar plates (nutrient agar with 1% [w/v] non-fat powdered milk) [14] and incubated at 37°C for 48 h. The diameter of the resulting clear zones was measured in triplicate using a digital caliper (Vernier Caliper DL3944, China). The proteolytic index (PI) value was calculated as follows:


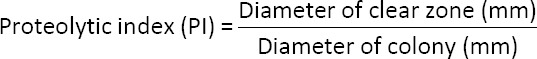


The selected strains with the highest PI value on skim milk agar were also screened for extracellular protease production in ultra-high temperature (UHT) skim milk using a modified method described by El-Ghaish *et al*. [14]. Selected strains were inoculated in MRS or M17 broth containing 0.05% (w/v) L-cysteine and incubated at 37°C for 24 h. The cell suspension was centrifuged at 5,000 × *g* for 10 min at 4°C using a Microfuge 20R Centrifuge (Beckman Coulter Inc., Brea, CA, USA), and then washed twice in Na-phosphate buffer (NaPB) (100 mM, pH 7.0), followed by resuspension in the same buffer solution. Subsequently, 50 μL of this was inoculated into 950 μL of UHT skim milk (EWEN, Hamburg, Germany) and incubated for 24 h. The control was prepared using an equal volume (50 μL) of NaPB solution in place of the inoculum. Hydrolysis of cow’s milk proteins by LAB strains was analyzed using sodium dodecyl sulfate-polyacrylamide gel electrophoresis (SDS-PAGE), and the results were explained by comparing the reductions in the intensity of the colored bands present in each sample.

### Proteolytic activity in the non-proliferative cell system

The proteolytic activity of the confirmed isolates was further assessed using a modified method involving non-proliferative cells, as described by El-Ghaish *et al*. [14], with Na-caseinate and denatured whey protein fractions as substrates. To prepare the substrates, Na-caseinate (Sigma) and whey proteins (Shanghai Yuanye Biotechnology Co. Ltd., Shanghai, China) were solubilized separately in NaPB and brought to a concentration of 10 mg/mL. The whey protein solution was denatured by heating at 80°C for 30 min in a water bath (Jinghong Technology Ltd., Shanghai, China). In this assay, an aliquot (200 μL) of overnight culture was applied on the surface of milk-citrate-agar plates to induce proteolytic enzyme production with the following composition: Skim milk powder 4.4 (%), Na-citrate 0.8 (%), yeast extract 0.1 (%), glucose 0.5 (%), and agar 1.5 (%) in 100 mL distilled water pH 7.0 [15]. After 24 h of incubation at 37°C, 1 mL of sterile saline solution (0.85% w/v NaCl, 10 mM CaCl_2_) was added to the plates, and the cells were collected with a sterile inoculating loop from the surface of the plates. The cell suspensions were washed twice in the same diluent, resuspended in NaPB to a final optical density (OD) of 20 at 600 nm (OD600), and kept at 37°C for 30 min to allow amino acid starvation [16]. Proteolytic activity was assessed by mixing different milk protein fractions (1:1, v/v) with cell suspensions, followed by incubation. Controls were prepared using an equal volume of NaPB in place of the inoculum, as previously described. At the end of incubation, the cell suspension was centrifuged at 4°C for 5 min at 4,000 × *g*. The supernatants were used to determine protein hydrolysis by SDS-PAGE.

### SDS-PAGE analysis

To determine the extent of protein hydrolysis, supernatants containing hydrolyzed proteins were analyzed in a vertical electrophoresis system (Mini-PROTEAN II Electrophoresis Cell, Bio-Rad Laboratories, USA) according to the method of Mao *et al*. [17] using polyacrylamide gel (12% resolving gel and 5% stacking gel). A pre-stained protein molecular weight marker (PageRuler^,^ Prestained Protein Ladder, 6.5–200 kDa; Thermo Fisher Scientific, Waltham, MA, USA) was loaded alongside the samples. After running the stacking gel at 80 V and the resolving gel at 150 V, the gels were stained with standard stain buffer (Shanghai Sangon Biotechnology Co. Ltd., Shanghai, China). The stained gels were washed with a destaining solution containing 40% (v/v) methanol and 10% (v/v) glacial acetic acid until the gel background was transparent. Gels were scanned using a FluorChem HD2 gel image system (ProteinSimple, Santa Clara, CA, USA), and the intensity of the bands was quantified using ImageJ software version 1.53a (National Institutes of Health, Bethesda, MD, USA). Analysis was performed using AlphaView SA software (ProteinSimple, Minneapolis, MN, USA). Protein hydrolysis was assessed by comparing the reduction in band intensity of each sample with that of the non-hydrolyzed control.

### Identification of proteolytic LAB strains

Proteolytic LAB strains were identified using phenotypic and genotypic analyses. Both biochemical and physiological properties were tested according to Bergey’s Manual of Systematic Bacteriology, Vol. 2 [18]. The genotypic characteristics were confirmed using 16S ribosomal RNA (*16S rDNA*) gene sequencing. The selected strains were grown in MRS or M17 broth containing 0.05% (w/v) L-cysteine, followed by incubation and centrifugation at 4°C for 10 min at 5,000 × *g*. Bacterial cells were harvested for genomic DNA extraction using bacterial genomic DNA extraction (Sangon Biotechnology Co. Ltd.). The universal primers 27F and 1492R were used to amplify the *16S rDNA* gene sequence [19]. The *16S rDNA* gene was amplified using a Bio-Rad polymerase chain reaction (PCR) cycler (Hercules, CA, USA) with a primary heating step for 3 min at 95°C, followed by 35 cycles of denaturation for 15 s at 95°C, annealing for 15 s at 60°C, and extension for 5 min at 72°C. The amplified PCR products were sequenced on an ABI 3730xl DNA Analyzer (Applied Biosystems, CA, USA) at Sangon Biotech (Shanghai, China). The obtained partial *16S rDNA* gene sequence (1,500 bp) of the selected strain was subjected to a homology search with the standard 16S rDNA sequences in the GenBank database using the basic local alignment tool (BLAST; National Center for Biotechnology Information, U.S. National Library of Medicine, National Institutes of Health, USA) [20] available in the NCBI website (http://www.ncbi.nlm.nih.gov). Multiple sequence alignments were performed using ClustalW v2.1 (European Bioinformatics Institute, EMBL-EBI, UK) with default parameters, and gaps were edited using BioEdit v7.0.5 (Tom Hall, Department of Microbiology, North Carolina State University, USA; http://www.mbio.ncsu.edu/bioedit/bioedit.html). A phylogenetic tree was constructed using MEGA version 7 (Molecular Evolutionary Genetics Analysis software; developed by Sudhir Kumar, Arizona State University, USA, and Koichiro Tamura, Tokyo Metropolitan University, Japan; http://www.megasoftware.net/mega.php) with the unweighted pair group method with arithmetic mean algorithm and a bootstrap analysis based on 1,000 replications.

### *In vitro* evaluation of probiotics and safety properties

#### Detection of virulence-related genes

To assess the safety of proteolytic LAB strains, the presence of virulence-related genes was examined. Targeted virulence-related genes included collagen adhesion (ace), aggregation substance (asa1), cytolysin structural subunits (cylLL and cylLS), cell wall adhesion (efaAfs), enterococcal surface protein (esp), gelatinase (gelE), and hyaluronidase (hy). PCR products were analyzed by electrophoresis on a 1% agarose gel. Genomic DNA from *Enterococcus faecalis strains* KT2W2G and CM6CR07 was used as a positive control [21].

#### Survival under simulated human gastrointestinal tract

To evaluate the survival through the human gastrointestinal tract, an *in vitro* GI transit assay was conducted according to the method of Naissinger da Silva *et al*. [22] with a slight modification (gastric juice was prepared by dissolving pepsin in PBS adjusted to pH 3.0 with HCl, instead of pepsin in HCl solution; intestinal juice contained ox gall bile and pancreatin adjusted to pH 8.0). Artificial gastric juice was prepared by dissolving 3.0 g/L of pepsin (Sigma) in PBS (50 mM, pH 7.0) and adjusting the pH to 3.0 using concentrated HCl. Simulated intestinal juice was prepared by mixing PBS with 0.3% (w/v) Ox-gall bile and 3.0 mg/mL pancreatin (Sigma) and adjusted the pH to 8.0 with 1 N NaOH. Both solutions were filter-sterilized using 0.22 μm syringe filters (Merck-Millipore, CA, USA). An aliquot (1 mL) of the overnight culture was centrifuged at 5,000 × *g* for 10 min at 4°C to determine the transit tolerance, and then the pellets were washed twice with sterile PBS solution (50 mM, pH 7.0). The washed cells were resuspended in 1 mL of artificial human gastric juice and incubated with mild shaking at 37°C for 3 h in a digital shaking incubator (model MQD-S3R, Shanghai Minquan Instrument Co., Ltd., Shanghai, China). After incubation, the cell suspension was centrifuged at 5,000 × *g* for 10 min at 4°C and resuspended in 1 mL of the simulated intestinal juice. After incubation, the cell suspension was centrifuged at 5,000 × *g* and for 10 min at 4°C, and then resuspended in 1 mL of simulated intestinal juice. The tubes were incubated at 37°C for 6 h in a shaking incubator with mild shaking. The cell viability of the selected strains was determined using a pour plate technique, in which a 1-mL aliquot of each dilution was cultured on MRS or M17 agar plates at 0 h (N0, representing the initial cell numbers before exposure to artificial gastric and intestinal juices) and after gastrointestinal fluid exposure (N_1_). The survival rate was calculated using the following equation:

Survival rate (%) = (Log CFU N_1_/Log CFU N_0_) × 100.

Where N_1_ and N_0_ are the total viable counts of the strains after and before exposure to artificial gastric and intestinal juices, respectively.

#### Hemolytic activity

The hemolytic activity was assessed as described by Suraporn *et al*. [23]. Fresh proteolytic LAB strains were streaked on Columbia agar plates (Oxoid Ltd., Basingstoke, UK) supplemented with 5% (v/v) defibrillated sheep blood. All plates were incubated at 37°C for 48 h. Hemolytic activity was determined based on the appearance of zones around colonies, including green zones (α-hemolytic or partially hemolytic), clear zones (β-hemolytic or completely hemolytic), and absence of zones (γ-hemolytic or non-hemolytic) on Columbia blood agar plates.

#### Antibacterial activity

The antibacterial activity of the selected proteolytic LAB strains against gut and food-borne pathogens was assessed using the soft-agar overlay technique described by Fagheei Aghmiyuni *et al*. [24]. Each overnight culture was adjusted in PBS to a final concentration of 10^8^ colony-forming units (CFU)/mL. Cell suspensions (10 μL) were spotted onto MRS or M17 agar plates containing 15 mL of culture medium. All plates were incubated for 24 h after a 10-minute drying period. The indicator strains used in this study, including *Escherichia coli* American Type Culture Collection (ATCC) 25922, *Staphylococcus aureus* ATCC 25923, *Listeria monocytogenes* ATCC 7644, and *Salmonella enterica* Serovar Typhi, were inoculated in tryptic soy broth and incubated at 37°C in a shaker incubator (New Brunswick Innova 44, Eppendorf AG, Hamburg, Germany) at 150 rpm for 4 h. The culture broths were adjusted to 0.5 McFarland scale (1.5 × 10^8^ CFU/mL) with sterile normal saline using a DEN-1 McFarland Densitometer (Grant Instruments Ltd., Cambridge, UK) and then diluted with 7 mL of soft tryptic soy agar (0.75% [w/v] agar) to obtain the final concentration of 10^6^ CFU/mL. Cell suspensions were overlaid onto MRS or M17 agar plates pre-inoculated with the selected proteolytic LAB strains. Plates were incubated, and inhibition zones were measured to assess antimicrobial activity.

#### BSH activity

The BSH activity was determined using a modified plate assay based on the method of Gebre *et al*. [25] with a slight modification. Briefly, overnight cultures of each proteolytic LAB strain (10 μL) were spotted onto BSH medium prepared by supplementing MRS or M17 agar with 0.37 g/L CaCl_2_ and 0.5% (w/v) of each bile salt: Sodium taurocholate hydrate, sodium taurodeoxycholate (TDC) hydrate, and sodium glycocholate (GC) hydrate (Sigma). The agar plates were used as a control, without supplementation with human bile salts. All plates were incubated at 37°C for 72 h. The presence of an opaque halo or granular white colonies was a positive result.

#### Antibiotic susceptibility

The antibiotic susceptibility of the proteolytic LAB strains was evaluated using the agar disc diffusion method described by Palladini *et al*. [26]. Each overnight culture was adjusted to 10^6^ CFU/mL with sterile normal saline solution. Cell suspensions were swabbed in three directions onto 4-mm-thick MRS or M17 agar (25 mL per dish) using a sterile cotton swab. After plate drying for 5 min, 15 different antibiotic discs (Oxoid, Basingstoke, United Kingdom), namely, 10 μg ampicillin (AMP10), 10 μg bacitracin, 75 μg cefoperazone (CPR75), 30 μg ceftazidime, 30 μg cephalothin (CLT30), 30 μg chloramphenicol (CHL30), 15 μg erythromycin (ERY15), 10 μg gentamicin, 30 μg kanamycin, 10 μg norfloxacin, 10 μg penicillin G (PEN10), 300 μg polymyxin B, 10 μg streptomycin, 30 μg tetracycline, and 30 μg vancomycin were placed aseptically and then incubated for 24 h. The antibiotic susceptibility was determined by measuring the inhibition zone diameter (mm). The results were interpreted as resistant (R), intermediate (I), and sensitive (S) based on the Clinical and Laboratory Standards Institute (CLSI) guidelines, document M100, 30^th^ edition [27].

### Statistical analysis

Data were assessed for normality (Shapiro-Wilk test) and homogeneity of variances (Levene test). One-way analysis of variance was used to evaluate group differences, with Tukey’s multiple range test used for *post hoc* pairwise comparisons. All statistical analyses were conducted using the Statistical Package for the Social Sciences (SPSS) v17.0 (SPSS Inc., Chicago, IL, USA). Statistical significance was set at p < 0.05. All experiments were performed in triplicate.

## RESULTS

### Isolation and screening of proteolytic LAB

LAB strains were isolated using MRS and M17 media under both aerobic and anaerobic conditions to capture the diversity of the LAB population. MRS agar was employed to isolate *Lactobacillus* spp., while M17 agar was used for *Streptococcus* spp. and *Lactococcus* spp. [28]. A total of 76 isolates were obtained from 34 samples, including dairy products, fermented vegetables, and fermented fish. All isolates were Gram-positive and catalase-negative, confirming their LAB identity. Based on cell morphology, lactobacilli accounted for 48.68% (37 isolates) and cocci for 51.32% (39 isolates).

**Figure 1:**
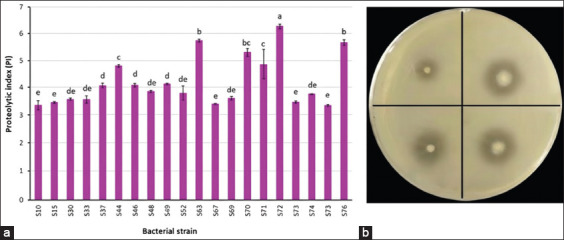
The ratio of the diameter of the clear zone to the diameter of the colony of the proteolytic production produced by the different isolates (a) and the appearance of clear zones surrounding the colony on S63 agar plates (b) after incubation at 37°C for 24 h.

Proteolytic activity screening on skim milk agar revealed that 70 out of 76 isolates exhibited proteolytic activity. The PI, calculated as the ratio of clear zone diameter to colony diameter, was used to determine the most active strains. Among these, 20 isolates showed high PI values ranging from 3.30 to 5.60 (Figure 1a). Notably, isolates S63, S72, and S76 exhibited the highest PI values of 5.59, 6.12, and 5.55, respectively.

### Proteolytic activity evaluation through SDS-PAGE

**Figure 2:**
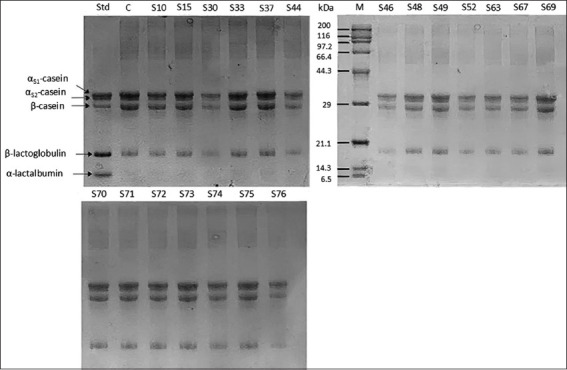
Sodium dodecyl sulfate-polyacrylamide gel electrophoresis showing the ultra-high temperature (UHT) skim milk hydrolysis profiles caused by different proteolytic strains after incubation at 37°C for 24 h. STD- = standard cow’s milk algens and C- = negative control (UHT skim milk without inoculating proteolytic strains).

Clear zones on skim milk agar confirmed proteolytic enzyme production [29] (Figure 1b). The proteolytic capacity of the 20 high-PI isolates was further assessed by incubating UHT skim milk (950 μL) with 50 μL of culture (~10^8^ CFU/mL) at 37°C for 24 h. Hydrolysis of milk proteins was then evaluated using SDS-PAGE (Figure 2).

Qualitative SDS-PAGE results demonstrated that isolates S30, S44, S46, S52, S63, S67, and S76 exhibited strong proteolytic activity across multiple milk protein fractions compared with the non-hydrolyzed control. Significant hydrolysis was defined as ≥50% reduction in band intensity relative to the control. Among milk fractions, β-LG was the most extensively degraded, particularly by isolates S30, S44, S46, and S76 (Figure 2).

### Hydrolysis of casein and whey proteins

**Figure 3:**
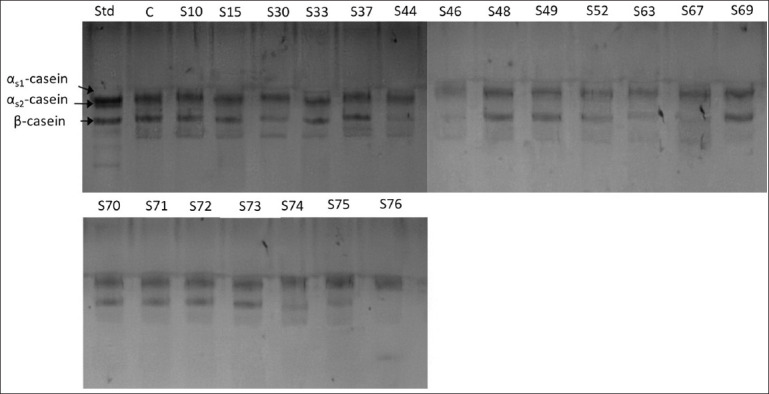
Sodium dodecyl sulfate-polyacrylamide gel electrophoresis showing the Na-caseinate hydrolysis profiles caused by different proteolytic strains after incubation at 37°C for 24 h. STD- = standard cow’s milk allergens, and C- = negative control (Na-caseinate without inoculating proteolytic strains).

Na-caseinate hydrolysis confirmed that isolates S30, S44, S46, S52, S63, S67, and S76 exhibited high activity against casein fractions compared with the control (Figure 3). Specifically, isolates S30, S44, S46, S67, and S76 showed strong degradation of β-CN.

**Figure 4:**
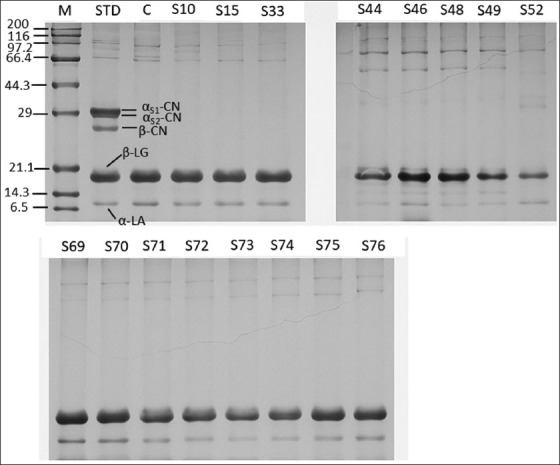
Sodium dodecyl sulfate-polyacrylamide gel electrophoresis showing the whey protein hydrolysis profiles caused by different proteolytic strains after incubation at 37°C for 24 h. STD- = standard cow’s milk allergens, and C- = negative control (whey protein without inoculating proteolytic strains).

The ability of selected strains to hydrolyze denatured whey proteins was also evaluated. Results showed that the same isolates (S30, S44, S46, S52, S63, S67, and S76) had higher proteolytic activity against β-LG than other isolates and the control (Figure 4). Among them, isolate S46 demonstrated the most pronounced β-LG degradation. Based on these findings, isolates S30, S44, S46, S48, S63, S67, S71, and S73 were selected for further analyses.

### Identification and phylogenetic analysis

The selected isolates were subjected to phenotypic and genotypic analyses. Morphological examination showed coccoid forms for S30 and S63, whereas S44, S46, S52, S67, and S76 were rod-shaped. All isolates grew in 5%–7% NaCl and tolerated pH 4.5–8.5 (Table 1).

**Table 1:** Biochemical and physiological properties of selected proteolytic LAB isolated from dairy products.

Property	Strain								

	S30	S44	S46	S48	S52	S63	S67	S71	S76
Shape	sphere	rod	rod	Sphere	rod	sphere	rod	rod	rod
Gram stain	+	+	+	+	+	+	+	+	+
Catalase test	−	−	−	−	−	−	−	−	−
Gas from glucose	−−	+	−	−	−	−	−	−	−
Growth at 15/45°C	−/+	+/+	+/+	−/+	+/+	−/+	+/−	+/−	+/+
Growth at pH 4.5/8.5	+/+	+/+	+/+	+/+	+/+	+/+	+/+	+/+	+/+
Growth at 5%, 6% and 7% NaCl	+	+	+	+	+	+	+	+	+
10% NaCl	+	−	+	+	−	+	−	−	+
Carbohydrate fermentation									
Amygdalin	−	−	−	−	+	−	+	+	+
Arabinose	−	+	−	−	+	−	−	−	−
Cellobiose	−	−	+	−	+	−	+	+	+
Esculin	−	−	+	−	+	−	−	−	+
Fructose	+	+	+	+	+	+	+	+	+
Galactose	+	+	+	+	+	+	+	+	+
Glucose	+	+	+	+	+	+	+	+	+
Lactose	+	+	+	+	+	+	+	+	+
Maltose	−	+	+	−	+	−	+	+	+
Mannitol	−	+	+	−	−	−	+	+	+
Raffinose	−	+	+	−	+	−	−	−	+
Rhamnose	−	−	+	−	+	−	−	−	+
Ribose	−	+	+	−	+	−	+	+	+
Sorbitol	−	+	+	−	+	−	+	+	+
Sucrose	+	+	+	+	+	+	+	+	+
Trehalose	−	−	+	−	−	−	+	+	+

+ = Positive reaction, − = Negative reaction, LAB = Lactic acid bacteria

**Figure 5:**
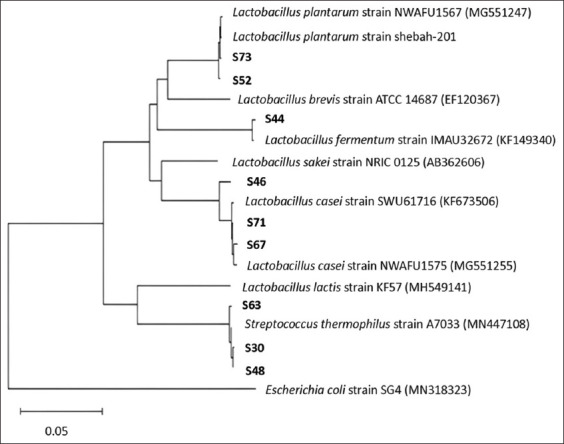
Phylogenetic tree showing the relationships between S30, S44, S46, S52, S63, and S67 isolates and LAB species based on *16S ribosomal RNA* gene sequences. The branching pattern was generated using the unweighted pair group method with arithmetic mean algorithm. The bootstrap values were based on 1,000 replicates. Bar, 0.03 substitutions per 100 nucleotide positions.

16S rDNA sequencing revealed that isolates S30 and S63 shared 99% similarity with *Streptococcus thermophilus* strain A7033 (GenBank: MN447108) (Figure 5). Other isolates were identified as *Lactobacillus fermentum* (S44), *Lactobacillus plantarum* (S52), and *Lactobacillus casei* (S46, S67, and S76). However, phenotypic traits of isolates S46 and S76 aligned more closely with *Lactobacillus rhamnosus* due to their ability to ferment rhamnose and grow at 45°C (Table 1) [30]. Isolate S67 was most consistent with *Lactobacillus paracasei* based on carbohydrate fermentation profiles, as it fermented lactose and sucrose but not rhamnose.

Thus, the identified strains included *S. thermophilus* (S30, S63), *L. plantarum* (S52), *L. rhamnosus* (S46, S76), *L. fermentum* (S44), and *L. paracasei* (S67). These isolates demonstrated the ability to hydrolyze major cow’s milk allergens. Moreover, *L. fermentum* S44, *L. rhamnosus* S46 and S76, and *L. paracasei* S67 achieved complete hydrolysis of β-CN.

### Evaluation of probiotic properties and safety

#### Detection of virulence-related genes

**Figure 6:**
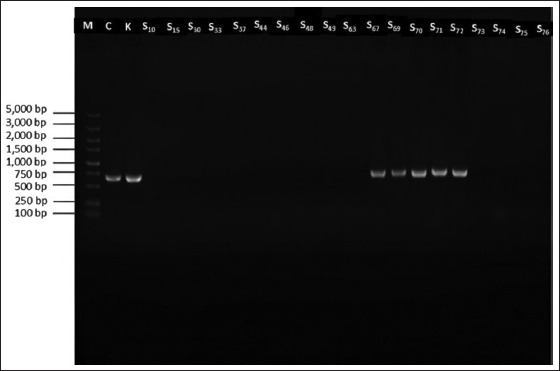
Presence of *efaAfs* virulence genes in different strains.

LAB are generally regarded as safe, but virulence-associated genes must be screened to avoid potential horizontal gene transfer [31, 32]. All strains tested negative for ace, asa1, age, cylLL, cylLS, esp, gelE, and hy (Table 2). For *L. paracasei* S67, all genes were absent except for efaAfs (Figure 6), which is associated with adhesion and has been linked to disease only in animal models. Given the absence of major virulence genes in all isolates, with the exception of efaAfs in one strain, all isolates were advanced for probiotic and safety evaluations.

**Table 2:** Presence of virulence genes in the selected proteolytic strains.

Proteolytic strain	Virulence gene								

	*asa1*	*ace*	*gelE*	*efaA_fs_*	*hyl*	*agg*	*esp*	*cylL_L_*	*cylL_S_*
S30	−	−	−	−	−	−	−	−	−
S44	−	−	−	−	−	−	−	−	−
S46	−	−	−	−	−	−	−	−	−
S48	−	−	−	−	−	−	−	−	−
S52	−	−	+	−	−	−	−	−	−
S63	−	−	−	−	−	−	−	−	−
S67	−	−	−	+	−	−	−	−	−
S71	−	−	−	+	−	−	−	−	−
S76	−	−	−	−	−	−	−	−	−

#### Survival under simulated GI tract conditions

**Figure 7:**
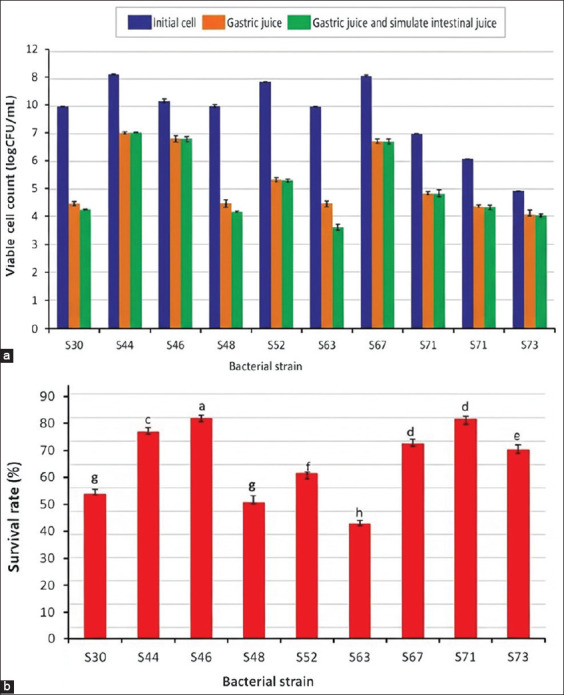
The Viable cell count (a) and survival rate (b) of proteolytic lactic acid bacteria strains in artificial human gastric juice incubated at 37°C for 3 h.

After 3 h exposure to gastric juice at pH 2.0, viable cell counts decreased by 2–4 log CFU/mL compared with initial levels (Figure 7a). *S. thermophilus* isolates S30, S48, and S63 exhibited poor gastric tolerance. However, all isolates survived well in intestinal juice. Notably, *L. rhamnosus* S46 showed the highest overall survival rate of 83.12% (6.76 log CFU/mL; p < 0.05) under simulated GI conditions (Figure 7b).

#### BSH and hemolytic activities

**Figure 8:**
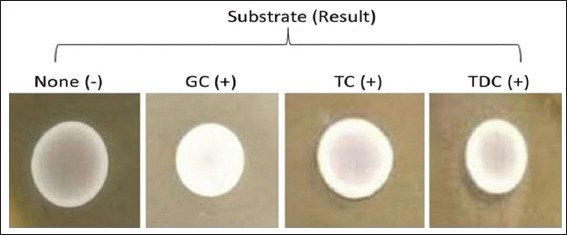
The presence of conjugated bile acids (glycocholate, taurocholic, and taurodeoxycholate) due to the precipitation of deconjugate bile salt or free bile acid forms.

BSH deconjugates bile salts, reducing cholesterol absorption [33]. BSH-positive strains formed opaque colonies in the presence of GC acid, taurocholic acid, and TDC acid (Figure 8) [34]. In this study, *L. fermentum* S44, *L. rhamnosus* S46, and *L. paracasei* S67 displayed BSH activity, while *L. rhamnosus* S76 did not (Table 3).

**Table 3:** Presence of bile salt hydrolase and hemolytic activity of proteolytic LABs.

Strain	Human bile salts			Haemolytic activity

	GC	TC	TDC	
*Streptococcus thermophilus* S30	−	−	−	−
*L. fermentum* S44	+	+	+	+
*Lactobacillus rhamnosus* S46	+	+	+	−
*L. plantarum* S52	−	+	+	−
*Streptococcus thermophilus* S63	−	−	+	−
*L. paracasei* S67	+	+	+	+
*Lactobacillus rhamnosus* S76	−	−	−	+

LAB = Lactic acid bacteria, GC = Glycocholate, TC = Taurocholic, TDC = Taurodeoxycholate

Hemolytic activity is a key safety consideration [35]. *L. fermentum* S44 exhibited α-hemolysis, while *L. paracasei* S67 and *L. rhamnosus* S76 showed β-hemolysis (Table 3). In contrast, *S. thermophilus* S30 and S63, *L. plantarum* S52, and *L. rhamnosus* S46 exhibited γ-hemolysis (no hemolysis) and were considered safe probiotic candidates. Strains exhibiting hemolytic activity warrant further genomic and *in vivo* validation before industrial application.

### Antimicrobial activity

LAB produce metabolites such as organic acids, hydrogen peroxide, diacetyl, and bacteriocins that inhibit pathogens [36, 37]. Antimicrobial activity was tested against four pathogens using soft-agar overlay. *L. rhamnosus* S46 and *L. plantarum* S52 produced the largest inhibition zones (Table 4). *S. thermophilus* S30 and S63 displayed only mild activity. *L. rhamnosus* S46 exhibited the strongest antimicrobial effect, with inhibition zones up to 35.59 mm against E. coli ATCC 25922 and *Salmonella Typhi*.

**Table 4:** The antimicrobial activity of proteolytic LABs against foodborne pathogens and food-spoilage microorganisms.

Indicator strain	Zone of inhibition (mm)			

	*Streptococcus thermophilus* S30	*Streptococcus thermophilus* S63	*Lactobacillus rhamnosus* S46	*Lactobacillus plantarum* S52
*Escherichia coli* ATCC25922	5.11 ± 0.04	7.38 ± 0.37	35.59 ± 0.37	25.45 ± 0.17
*Listeria monocytogenes* ATCC7644	6.62 ± 0.34	9.05 ± 0.16	35.59 ± 0.37	25.16 ± 0.22
*Staphylococcus aureus* ATCC25923	5.41 ± 0.23	8.42 ± 0.23	34.73 ± 0.16	24.50 ± 0.11
*Salmonella* Typhi	5.28 ± 0.27	7.79 ± 0.15	35.19 ± 0.15	27.04 ± 0.07

LAB = Lactic acid bacteria, ATCC = American type culture collection

### Antibiotic susceptibility

Antibiotic susceptibility was evaluated using disc diffusion against 15 antibiotics (Table 5). Breakpoints were interpreted according to CLSI [28, 38]: Inhibition zones ≤14 mm (resistant), 15–19 mm (intermediate), and ≥20 mm (sensitive). All proteolytic LAB strains were sensitive to AMP, CPR, CLT, CHL, ERY, and PEN. *S. thermophilus* S63 was the most sensitive overall, while *L. plantarum* S52 showed the highest resistance.

**Table 5:** Susceptibility of selected proteolytic LAB strains to antibiotics used.

Antibiotic	Inhibition zone (mm)			

	S30	S46	S52	S63
Ampicillin (10 mg, AMP10)	41 (S)	27 (S)	33 (S)	38 (S)
Bacitracin (10 mg, BAC10)	0 (R)	0 (R)	0 (R)	21 (S)
Cefoperazone (75 mg, CPR75)	32 (S)	32 (S)	31 (S)	56 (S)
Ceftazidime (30 mg, CTD30)	24 (S)	10 (R)	22 (S)	44 (S)
Cephalothin (30 mg, CLT30)	38 (S)	24 (S)	40 (S)	57 (S)
Chloramphenicol (30 mg, CHL30)	27 (S)	29 (S)	26 (S)	44 (S)
Erythromycin (15 mg, ERY15)	39 (S)	33 (S)	27 (S)	36 (S)
Gentamycin (10 mg, GEN10)	21 (S)	18 (I)	13 (R)	24 (S)
Kanamycin (30 mg, KAN30)	0 (R)	13 (R)	0 (R)	19 (I)
Norfloxacin (10 mg, NOR10)	16 (I)	12 (R)	0 (R)	31 (S)
Pencillin G (10 mg, PEN10)	43 (S)	27 (S)	32 (S)	33 (S)
Polymyxin B (300 mg, PMX300)	16 (I)	0 (R)	0 (R)	18 (I)
Streptomycin (10 mg, STR10)	20 (S)	22 (S)	15 (R)	11 (R)
Tetracycline (30 mg, TET30)	29 (S)	38 (S)	20 (S)	46 (S)
Vancomycin (30 mg, VAN30)	23 (S)	0 (R)	0 (R)	33 (S)

S = Susceptible, I = Intermediate, R = Resistant, LAB = Lactic acid bacteria

Because probiotics are administered as live organisms, the presence of transferable resistance genes is a safety concern [38]. Therefore, resistance traits observed in *L. plantarum* S52 highlight the need for genomic validation before commercial application.

## DISCUSSION

### Isolation and proteolytic potential of LAB

In this study, 76 LAB strains were isolated from traditional fermented foods, and seven isolates demonstrated strong proteolytic activity, particularly against β-LG and β-CN. These proteolytic capabilities are consistent with those reported for potent LAB strains isolated from fermented dairy products using advanced techniques such as genome analysis and high-throughput screening [39].

### Identification and phenotypic characteristics

The top-performing isolates were identified as *S. thermophilus, Lactobacillus rhamnosus*, *L. plantarum, L. paracasei*, and *L. fermentum* based on 16S rDNA sequencing. This finding aligns with previous reports by Meng *et al*. [40] and Lee *et al*. [41], which demonstrate the effectiveness of these species in milk protein hydrolysis. Moreover, these isolates tolerated salt concentrations of up to 7% and acidic environments with a pH as low as 4.5, a characteristic consistent with the findings of He *et al*. [42] for LAB from fermented dairy products.

### Safety and probiotic properties

Safety evaluations produced encouraging results. *L. rhamnosus* S46 and *L. plantarum* S52 survived simulated gastrointestinal conditions, lacked major virulence genes, exhibited no hemolytic activity, and remained sensitive to clinically relevant antibiotics. These traits fulfill the criteria recommended by Food and Agriculture Organization/World Health Organization for probiotic strains [43].

### Functional traits and health relevance

The functional characteristics of isolates S46 and S52 support their potential as functional starter cultures. Their strong milk protein-degrading ability enables natural allergen reduction without reliance on exogenous enzymes, meeting consumer demands for clean-label dairy (CLD) products [44]. In addition, *L. rhamnosus* S46 displayed robust BSH activity and broad-spectrum antimicrobial effects, attributes previously linked to cholesterol reduction and improved gut health in animal models [45, 46]. Both strains also survived simulated digestion at viable counts sufficient to confer probiotic benefits.

#### One Health perspective

The findings of this study have significant implications within the One Health framework, which emphasizes the interconnectedness of human, animal, and environmental health. CMA not only affects human health but also influences dietary practices, economic sustainability in the dairy industry, and consumer trust in food safety. By identifying LAB strains such as *L. rhamnosus* S46 and *L. plantarum* S52 with strong allergen-degrading capacity, probiotic functionality, and safety attributes, this research contributes to safer dairy production systems that benefit both consumers and industry.

From a public health perspective, the natural reduction of allergenic milk proteins through fermentation provides a sustainable alternative to chemical or high-cost industrial methods, reducing the risk of allergic reactions in sensitive populations, particularly children. In terms of food safety, the antimicrobial properties of the selected LAB strains further enhance product quality by inhibiting foodborne pathogens, thereby supporting consumer protection. Finally, the demonstrated BSH activity of these isolates may contribute to improved gut health and cholesterol metabolism, aligning with the broader One Health goals of reducing chronic disease risks and promoting nutritional well-being.

Thus, integrating such multifunctional LAB strains into dairy production not only addresses a critical food allergy problem but also supports a holistic approach to health that connects safe food systems, sustainable industry practices, and improved human well-being.

## CONCLUSION

This study successfully isolated 76 LAB strains from traditional fermented foods, of which seven exhibited strong proteolytic activity, particularly against β-LG and β-CN. Among these, *L. rhamnosus* S46 and *L. plantarum* S52 demonstrated the most promising functional attributes, including high allergen-degrading ability, survival under simulated gastrointestinal conditions, absence of major virulence genes, lack of hemolytic activity, sensitivity to clinically important antibiotics, and probiotic-associated traits such as BSH activity and antimicrobial effects.

These findings highlight the potential of *L. rhamnosus* S46 and *L. plantarum* S52 as functional starter cultures for the development of hypoallergenic, probiotic-enriched dairy products. Their natural proteolytic activity offers a safe and consumer-acceptable alternative to industrial enzymatic treatments, aligning with the growing demand for CLD products. In addition, their BSH activity and antimicrobial effects suggest added health benefits, including cholesterol reduction and gut microbiota modulation, contributing to both food safety and One Health goals.

A major strength of this research is its comprehensive evaluation framework, integrating proteolytic activity, probiotic functionality, and safety profiling. The study combined both phenotypic and genotypic characterization, ensuring accurate identification and reliable functional assessment of LAB strains.

This investigation was limited to *in vitro* analyses under laboratory conditions, which may not fully reflect strain performance in complex dairy matrices or *in vivo* environments. Furthermore, while antibiotic susceptibility testing identified no immediate safety concerns, the potential for horizontal gene transfer of resistance determinants cannot be excluded without whole-genome sequencing.

Future research should include pilot-scale dairy fermentation trials to evaluate allergen reduction in real products, sensory and storage stability, and consumer acceptance. *In vivo* studies and genomic-level assessments are also required to confirm probiotic efficacy, cholesterol-lowering potential, and long-term safety before commercial application.

In summary, this study identifies *L. rhamnosus* S46 and *L. plantarum* S52 as safe, effective, and multifunctional LAB strains with potential application in the dairy industry. Their integration into industrial fermentation processes could support the production of safer, allergen-reduced dairy products, offering tangible health benefits to individuals with CMA while advancing the broader objectives of food safety, public health, and One Health sustainability.

## DATA AVAILABILITY

All data generated during the study are included in the manuscript.

## AUTHORS’ CONTRIBUTIONS

JC: Supervised the study. JC, CW, and LF: Conceptualized and performed the study and drafted and revised the manuscript and JST and LRS: Performed statistical analysis. JC, CW, LF, JST, and LRS: Interpreted the results and drafted and revised the manuscript. All authors have read and approved the final manuscript.
